# Acute Moderate-Intensity Exercise Generally Enhances Attentional Resources Related to Perceptual Processing

**DOI:** 10.3389/fpsyg.2019.02547

**Published:** 2019-11-08

**Authors:** Fangyuan Zhou, Chaoling Qin

**Affiliations:** College of Physical Education, Institute of School Sports Development, Southwest University, Chongqing, China

**Keywords:** acute exercise, cognitive function, ERP, P200, N200

## Abstract

The present study aimed to investigate whether acute moderate-intensity exercise led to a selective effect on executive function tasks or general effect on cognitive tasks that involve executive function and basic information processing in young adults. Besides, we also aimed to examine acute exercise’s effect on multiple ERP components (e.g., P2, N2, P3b, and N450) to expand previous research. Seventy-two young adults were randomly assigned to the exercise or control groups. The Stroop task was administrated before and after treatments (exercise or reading), and the P2, N2, P3b, and N450 components of the Event-Related Potential (ERP) waveform were recorded and analyzed. Larger P2 amplitudes on both congruent and incongruent tasks were observed following acute exercise. Acute exercise did not influence accuracy or response time, and no effects on N2, P3b, and N450 components were found. These findings suggest that acute moderate-intensity exercise may have a generally beneficial effect on mobilization of attentional resources related to perceptual processing and exercise-related physiological arousal.

## Introduction

A number of studies indicate that there is a positive effect of acute exercise on cognitive performance (Chang et al., 2012; Gajewski and Falkenstein, 2016; Kao et al., 2019). While two hypotheses give different opinions about the effect of acute exercise on cognitive function. A “general improvement hypothesis” indicates that acute exercise leads to general promotional effects on cognitive tasks that involve executive function (e.g., incongruent task during the Stroop task) and basic information processing (e.g., congruent and neutral tasks during the Stroop task) (Lichtman and Poser, 1983; Chang et al., 2014a, b). For example, Chang et al. (2014a) found acute moderate-intensity exercise for 25 min improved cognitive performance on both congruent and incongruent Stroop tasks in old adults. However, a “selective improvement hypothesis” suggests that acute exercise results in larger effects for executive function tasks that require more extensive cognitive demand than basic information processing (Hillman et al., 2003; McMorris and Hale, 2012). For example, a meta-analysis found acute moderate-intensity exercise showed a larger effect size on executive tasks than recall and alertness/attention tasks (McMorris and Hale, 2012).

The two different hypotheses about acute exercise’s effects on cognitive function may be related to methodologies employed in previous studies such as the cognitive task type (e.g., the Stroop task, the flanker task), exercise modality (e.g., aerobic exercise, resistance exercise), exercise duration (e.g., from 15 to 45 min), exercise intensity (e.g., moderate-intensity, high-intensity), and the selected populations (e.g., younger adults, older adults) (Chang et al., 2012).

Executive function (also known as cognitive control) refers to metalevel and higher cognitive processes that are necessary for goal-directed and achieving purposeful behaviors, especially in non-routine circumstances (Alvarez and Emory, 2006). Executive function is usually measured by the Stroop task in experimental situations. In the Stroop task, participants are asked to discriminate the colors in which stimulus words are written. When a word and the color in which it is written are incongruent (e.g., when the word “YELLOW” is written in green), this conflict will result in increased error rates and response times. The incongruent task involves several executive functions, including inhibition, selective attention and cognitive flexibility (Liotti et al., 2000; Buck et al., 2008). Improvement of performance on the Stroop task may result from the facilitation of processing of relevant information or suppression of the irrelevant stimuli (inhibition) (Neill et al., 1995). To investigate the cerebral basis of cognitive function, the high temporal resolution of ERPs is particularly suitable. A standard ERP to a visual stimulus contains a sequence of components, with earlier components (P1, N1, and P2) relating to the early information processes and later components (N2, P3b, and N450) relating to cognitive processes (Rugg and Coles, 1996). P3b component is centro-parietal positivity occurring 300–600 ms and usually considered to reflect performance in conflict resolution. P3b amplitude is associated with the amount of neural or attentional resources allocated to a given task (Polich, 2007) and P3b latency is related to the speed of stimulus classification and evaluation (Verleger, 1997). Drollette et al. (2014) found acute aerobic exercise led to decreased P3b latency for both congruent and incongruent flanker tasks in children. While O’Leary et al. (2011) found moderate-intensity aerobic exercise generally increased the allocation of attentional resources reflected by P3b amplitude during the flanker task in young adults. Similarly, Chang et al. (2017) found aerobic exercise led to a generalized improvement in attentional or neural resource allocation reflected by P3b amplitude during the Stroop task in young adults. With the growth of age, acute exercise maybe shows different effects on the P3b component.

Besides, most ERP studies about acute exercise’s effects on cognitive function have predominantly focused on the P3b component, only a few studies have focused on other ERP components such as P2, N2, and N450. An early frontal P2 occurring approximately 150–250 ms after stimulus onset is confirmed to be connected with perceptual processing which requires attention allocation to function (Yuan et al., 2007, 2012), and the P2 component is also extremely sensitive to the arousal level (Schaefer et al., 2011; Feng et al., 2014; Imbir et al., 2017). Kumar et al. (2010) found P2 latency generally decreased during an auditory oddball paradigm following the acute moderate exercise for 5 min in young adults. N2 is a fronto-central negativity component occurring 200–350 ms and also usually considered to reflect performance in conflict resolution tasks (Ligeza et al., 2018). N2 is connected with the conflict detection process (Chen et al., 2016; Li et al., 2017; Hu et al., 2019) and regarded as reflecting inhibition (Ligeza et al., 2018), the resolution of response conflict and response selection (Küper et al., 2017). Within conflict resolution tasks, a larger N2 amplitude is observed for conflict trials (e.g., incongruent Stroop task) than non-conflict trials, which is typically interpreted as evidence for inhibition of incorrect responses. Ligeza et al. (2018) found acute moderate-intensity exercise increased conflict effect of N2 amplitude during flanker task in young adults, which suggested acute exercise might directly benefit inhibition. While, Drollette et al. (2014) found acute moderate-intensity exercise led to smaller N2 amplitudes for both congruent and incongruent flanker tasks in preadolescent children, which suggested acute exercise might have a general effect on the conflict detection process. N450 which occurs about 350–500 ms after stimulus onset is sensitive to interference control in the Stroop task (West and Alain, 2000; Imbir et al., 2017). It has been proved that the N450 component reflects activation of the anterior cingulate cortex (Liotti et al.) possibly associated with conflict detection (West, 2003) or the selection of competing responses (West and Alain, 1999). Chang et al. (2017) found reduced N450 amplitudes and decreased N450 latencies for both congruent and incongruent Stroop tasks after acute moderate-intensity exercise in young adults. These evidences show acute exercise’s effects on other ERP components (e.g., N2) are still quite mixed, lots of work remains to be done to explore this problem.

In the present study, the purpose was to investigate whether acute moderate-intensity exercise led to a selective effect on executive function tasks or general effect on cognitive tasks that involve executive function and basic information processing in young adults. Besides, we also aimed to examine acute exercise’s effect on multiple ERP components (e.g., P2, N2, P3b, and N450) to expand previous research. Most previous studies (Kamijo et al., 2009; Chang et al., 2017; Ligeza et al., 2018) used an inter-group design to examine the effect of acute exercise on cognitive function. As is well known, this type of design only includes post-test measurements and may be susceptible to the effect of day-to-day variability. To overcome the drawback, we used a between-group design in our study, the Stroop task was administrated before and after exercise, and we added a control group to reconcile practice effects. Based on this, a 2 (Treatments: exercise vs. reading) × 2 (Time: pre-test vs. post-test) design was used in our study to examine the effect of acute exercise on cognitive function. Chang et al. (2017) found acute exercise led to reduced response times for both Stroop congruent and incongruent task conditions. While O’Leary et al. (2011) found decreased RT interference (Incongruent RT–Congruent RT) following acute exercise during the flanker task. And most studies found the beneficial effect of acute exercise on the P3b component (Kamijo et al., 2009; O’Leary et al., 2011; Chu et al., 2017). On the same basis, we hypothesized that acute exercise would benefit for response time and the P3b component.

## Materials and Methods

### Participants

Seventy-two College students (36 females) were recruited through advertisements at Southwest University in China. Participants must meet the following criteria: between the ages of 18 and 26 years, right-handedness, normal or corrected-to-normal vision and color perception, a body mass index (BMI) less than 25, and no psychiatric, neurological disorders, cardiovascular disease or physical disability. To overcome the ceiling effect of exercise, participants must have no exercise habits [the criteria of exercise habits are to exercise at least 3 times a week, each time over 30 min at moderate exercise intensity (heart rate >110 times/min), and maintain for more than a year]. Participants completed the Physical Activity Readiness Questionnaire (PAR-Q) (American College of Sports Medicine, 2006), the Edinburgh handedness inventory (Oldfield, 1971), the international physical activity questionnaire (IPAQ) (Hagströmer et al., 2006), and the shortened version of Raven’s Advanced Progressive Matrices test (sRAPM) (Marzecová et al., 2013). *A priori* power analysis revealed that 66 participants were needed to observe a moderate effect of experimental manipulation (see section Power calculations). The three additional participants for each group were invited to the study in case of participant dropout or EEG data collection failures. Participants were randomly assigned to the exercise or control groups, in which each group has the equal number of males and females (see section randomly group). Participants were asked to refrain from exercise before 24 h, and wear comfortable clothes and shoes to attend the exercise in the experiment. All participants were paid 60 RMB (about nine dollars) for their participation and signed informed consent. Participants’ characteristics are summarized in Table 1. This study was approved by the local Review Board for Human Participant Research, and each participant signed an informed consent form before the experiment. This study was conducted following the ethical principles of the Helsinki Declaration regarding human experimentation (World Medical Organization, 1996).

**TABLE 1:** Descriptive data for participants’ demographics between two groups (mean ± SE).

	**Experiment group**	**Control group**	**Total**
	**(*n* = 36)**	**(*n* = 36)**	**(*n* = 72)**
Age (years)	20.25 ± 0.21	19.89 ± 0.21	20.07 ± 0.15
Height (cm)	167.14 ± 1.39	168.28 ± 1.45	167.71 ± 1.00
Weight (kg)	55.97 ± 1.39	58.94 ± 1.21	57.46 ± 0.93
BMI (kg/m2)	19.96 ± 0.32	20.78 ± 0.28	20.37 ± 0.22
HRmax (bpm)	199.75 ± 0.21	200.11 ± 0.21	199.93 ± 0.15
IPAQ (MET)	1556.69 ± 160.56	1672.28 ± 145.57	1614.49 ± 107.82
IQ^a^	12.13 ± 0.22	12.49 ± 0.22	12.31 ± 0.16

*BMI, body mass index; HRmax, maximum heart rate; HRmax = 220-age; IPAQ, international physical activity questionnaire; MET, metabolic equivalent. ^*a*^As measured by a shortened version of Raven’s Advanced Progressive Matrices test (sRAPM).*

### Stimuli

The present study used a Stroop task. The experiment used only one block that was made up of two types of tasks: congruent and incongruent. Congruent trials were composed of one of four color words printed in Chinese and in the same color [i.e., 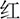 (RED), 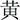 (YELLOW), 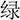 (GREEN) or 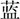 (BLUE) in the color of red, yellow, green or blue, individually]. Incongruent trials were composed of identical four-word colors but printed in different colors [e.g., 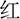 (RED) printed in blue ink color]. The block was composed of 120 trials consisting of 60 congruent and 60 incongruent trials, and the presentation of trials was random.

### Procedures

**FIGURE 1:**
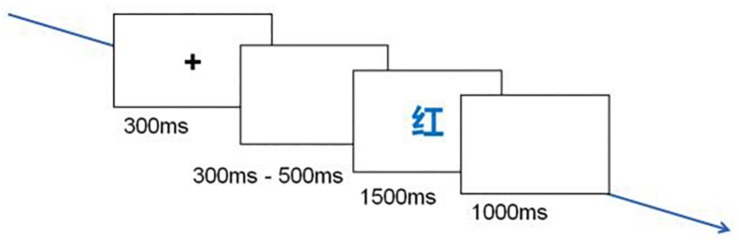
Schematic illustration of the behavioral procedure in a trial.

Step1: Before the experiment, subjects were told that they would take part in a color-naming task. Subjects sat about 80 cm away from a computer screen in an aurally isolated room. They were asked to place their left middle, left index, right index and right middle fingers on the s, d, j and k keys, each of which stood for one color. Each trial began with the 300 ms presentation of a small, white cross in the center of a black display screen. Between 300 and 500 ms after the offset of the cross, a stimulus word written in one of four colors appeared. Subjects were required to discriminate the color in which the word was written as quickly and accurately as possible by pressing the key according to the color. Word presentation was terminated by a key press or after 1500 ms, whichever occurred first. Hence, responses had to be given within 1500 ms. A 1000 ms presentation of a blank screen followed each termination (Figure 1). A practice session consisting of 10 trials was used to allow subjects to build the mapping of response fingers and colors. The practice session repeated until an accuracy rate of 90% was reached. Then Subjects were allowed to participate in the experiment. Throughout the block, subjects were asked to keep their eyes fixated on the computer display screen.

Step2: When finishing the Stroop task, the participants were randomly divided into two groups: experiment and control groups. In the experiment group, a Huawei heart rate watch was equipped to measure the subjects’ heart rates, the resting heart rate was measured within 3 min following the Stroop task. Then participants pedaled on the cycle for a 25-min session that consisted of 3-min warm up, 20-min steady-state exercise performed at 60–70% of Maximum Heart Rate, and 2-min cool down. Throughout the experimental treatment session, heart rate and RPE were recorded every 2 min. Then participants were asked to rest until their heart rates returned to within 110% of their resting hearts, this process took about 15 min. In the control group, participants were asked to read an article describing the scenery for 25 min, and then sit quietly and rest for 15 min.

**FIGURE 2:**
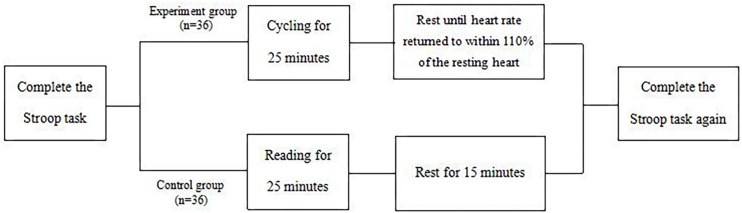
The flowchart of procedures.

Step 3: All subjects completed the Stroop task again. Figure 2 provides a flowchart of procedures.

### ERP Recording and Analysis

Continuous electroencephalographic (EEG) was recorded from 32 scalp sites placed according to the international 10–20 system using an elastic cap (Neuroscan). The montage was referenced offline to the average of right and left mastoids, and electrodes were initially referenced online to the Common Mode Sense electrode located at the CPz electrode. The EEG was amplified using a DC-100 Hz bandpass and continuously sampled at 250 Hz/channel. Impedance of all electrodes was maintained below 10 KΩ.

ERP data were analyzed using EEGLAB software (Delorme and Makeig, 2004). Averaging of ERPs was computed off-line and a 0.1–30 Hz filter was used. EEG data were segmented offline in epochs of 1,200 ms, with the 200 ms pre-stimulus period used for baseline correction. Eye movement artifacts (blinks and eye movements) were corrected using run Independent Component Correlation Algorithm (runICA) in the EEGLAB (Delorme and Makeig, 2004). On average, approximately 5% of components for each participant were removed. For each condition, the mean number of remaining components was as follows (mean ± SE): Pre-test of experiment group (30.40 ± 0.10); Post-test of experiment group (30.49 ± 0.10); Pre-test of control group (30.64 ± 0.10); Post-test of control group (30.53 ± 0.09). Trials with artifacts due to amplifier clipping or peak-to-peak deflection exceeding ±100 μV were excluded from averaging, and trials with response errors were also rejected. On average, approximately 13% of trials for each participant were removed. For each condition, the mean number of remaining trials after behavioral correctness analysis and EEG artifact rejection was as follows (mean ± SE for congruent/incongruent trials): Pre-test of experiment group (53.74 ± 0.64/49.63 ± 0.79); Post-test of experiment group (54.71 ± 0.58/51.26 ± 0.71); Pre-test of control group (54.42 ± 0.63/51.42 ± 0.78); Post-test of control group (53.64 ± 0.58/50.39 ± 0.70). Stimulus-locked ERP averages which only included correct trials were computed individually for each participant for the congruent and incongruent tasks, separately. Consistent with previous studies (Feng et al., 2014; Chang et al., 2017; Imbir et al., 2017; Ligeza et al., 2018), visual inspection of the grand-averaged ERP waveforms (congruent vs. incongruent; difference wave) indicated that the P2 component emerged in the 160–200 ms time window at fronto-central scalp sites (i.e., Fz, F3, F4, FC1, and FC2 electrodes), the N2 component emerged in the 280–320 ms time window at fronto-central scalp sites (i.e., Fz, F3, F4, FC1, and FC2 electrodes), the P3b component emerged in the 360–420 ms time window at the centro-parietal scalp sites (i.e., CP1, CP2, Pz, P3b, and P4 electrodes), and the N450 component emerged in the 430–490 ms time window at fronto-central scalp sites (i.e., Fz, F3, F4, FC1, and FC2 electrodes). Thus, the P2, N2, P3b, and N450 components were measured as the difference waves between the congruent and incongruent tasks at the specified electrodes and time windows. Meanwhile, the topographic scalp distributions of the P2, N2, P3b, N450 components was illustrated based on all 32 electrode sites with spherical interpolation.

### Statistical Analysis

#### Power Calculations

Physical activity has a moderate effect on performance in conflict resolution tasks (Chang et al., 2012; Ligeza et al., 2018). *A priori* power analysis using G^∗^Power 3 software revealed that 66 participants were needed to observe a moderate effect (effect size *f* = 0.25) of experimental manipulation. The estimate was based on 80% power, 2 repeated measures from two groups and an alpha of 0.05 (Faul et al., 2007).

#### Randomly Group

SPSS v.17 (SPSS, Chicago, IL, United States) was used to randomly group. Males were numbered 1–36 according to the order of enrollment. Initially, a starting point was set in Random Number Generators, and then Rv.Uniform(0,1) in Compute Variable was used to generate a random number for each participant. Next, a cutpoint was set in Equal Percentiles Based on Scanned Cases in Visual Binning, and then males were randomly assigned to the experiment or control groups. Afterward, these operations were repeated to ensure females were randomly assigned to the experiment or control groups.

#### Behavioral and ERP Analyses

All analyses were conducted utilizing SPSS v.17 (SPSS, Chicago, IL, United States). An independent-samples *T*-Test was used to examine the discrepancy of participants’ characteristics between the experiment and control groups. A one-way repeated measures analysis of variance (ANOVA) was used to examine the effectiveness of the exercise treatment. A 2 (Treatments: exercise vs. reading) × 2 (Tasks: congruent vs. incongruent) × 2 (Time: pre-test vs. post-test) repeated measures ANOVA was used separately for accuracy and response time to examine the effect of exercise treatment on behavioral performance. For ERP measures, a 2 (Treatments) × 2 (Tasks) × 2 (Time) repeated measures ANOVA was used separately for amplitude and latency for the P2 component (at Fz in the 160–200ms time window), the N2 component (at Fz in the 280–320ms time window), the P3b component (at Pz in the 360–420 ms time window), and the N450 component (at Fz in the 430–490 ms time window) to examine the effect of acute exercise. Greenhouse-Geisser epsilon corrections were used when necessary to meet the assumption of sphericity. *Post hoc* comparisons were conducted with family-wise alpha levels set at 0.05 using Bonferroni significant difference tests.

## Results

### Demographic Analyses

As shown in Table 1, the experiment and control groups did not show significant difference in age, height, weight, BMI, HRmax (bpm), IPAQ (MET), and IQ, *t*(1,70) = 1.21, 0.57, 1.62, 1.93, 1.21, 0.53, 1.17, *p* = 0.23, 0.57, 0.11, 0.06, 0.23, 0.60, 0.25.

### Exercise Treatment Manipulation

In the experiment group, the one-way ANOVA revealed a significant main effect for time, *F*(35) = 934.35, *p* < 0.001, partial η^2^ = 0.96. Follow-up comparisons revealed that heart rate during exercise was significantly higher (132.94 ± 1.10 bpm) than post-treatment heart rate (75.56 ± 1.20 bpm) and resting heart rate (73.69 ± 1.26 bpm), *p*s < 0.001, partial η^2^ = 0.97, 0.98, whereas no significant difference was observed between post-treatment heart rate and resting heart rate, *p* = 0.76. Moderate-intensity exercise was defined as “somewhat hard” (13th point of the RPE scale) in Kamijo et al. (2004, 2009); 60–70% of HRmax in Drollette et al. (2014), the average heart rate (66.62% HRmax) and RPE (13.51) values assessed during exercise suggested that subjects exercised at moderate-intensity in the experiment group.

### Behavioral Data

Table 2 summarizes task performance values for accuracy and response time for each condition.

**TABLE 2:** Behavioral data (mean ± SE) across treatment and Stroop tasks.

**Behavioral data**	**Experiment group**				**Control group**			
	**Pre-test**		**Post-test**		**Pre-test**		**Post-test**	
	**Congruent**	**Incongruent**	**Congruent**	**Incongruent**	**Congruent**	**Incongruent**	**Congruent**	**Incongruent**
Accuracy (% © SE)	96.94 ± 0.60	90.88 ± 1.08	97.69 ± 0.54	95.65 ± 0.73	96.67 ± 0.60	90.65 ± 1.08	97.59 ± 0.54	94.49 ± 0.73
RT (ms © SE)	688.05 ± 13.88	822.37 ± 17.2	669.74 ± 11.82	781.81 ± 13.50	700.30 ± 13.88	810.43 ± 17.20	664.59 ± 11.82	776.53 ± 13.50

#### Accuracy

The three-way ANOVA revealed a significant main effect for time, *F*(1, 70) = 43.40, *p* < 0.001, partial η^2^ = 0.38, with post-test (96.35 ± 0.38%) yielding higher accuracy than pre-test (93.78 ± 0.52%). There was also a significant main effect for task, *F*(1, 70) = 76.30, *p* < 0.001, partial η^2^ = 0.52, with congruent task (97.22 ± 0.37%) yielding higher accuracy than incongruent task (92.92 ± 0.58%). And there was a significant interaction between task × time, *F*(1, 70) = 34.42, *p* < 0.001, partial η^2^ = 0.33. Follow-up analyses revealed that the accuracy of post-test (97.64 ± 0.38% and 95.07 ± 0.51%) was higher than pre-test (96.81 ± 0.43% and 90.76 ± 0.76%) for both congruent and incongruent trials, *p* = 0.019, *p* < 0.001, partial η^2^ = 0.08, 0.42. And the accuracy of congruent trials was higher than incongruent trials for both pre-test and post-test, *p*s < 0.001, partial η^2^ = 0.55, 0.29. No other significant main effect, two-way or three-way interactions were observed.

#### Response Time

The three-way ANOVA revealed a significant main effect for time, *F*(1, 70) = 23.63, *p* < 0.001, partial η^2^ = 0.25, with pre-test (755.29 ± 10.68 ms) yielding longer response time than post-test (723.17 ± 8.59 ms). There was also a significant main effect for task, *F*(1, 70) = 642.66, *p* < 0.001, partial η^2^ = 0.90, with incongruent task (797.78 ± 10.18 ms) yielding longer response time than congruent task (680.67 ± 8.54 ms). And there was a significant interaction among treatment × task × time, *F*(1, 70) = 4.40, *p* = 0.04, partial η^2^ = 0.06. Follow-up analyses revealed that the response time of pre-test (688.05 ± 13.88 ms, 822.37 ± 17.20 ms, and 700.30 ± 13.88 ms, 810.43 ± 17.20 ms) was longer than post-test (669.74 ± 11.82 ms, 781.81 ± 13.50 ms and 664.59 ± 11.82 ms, 776.53 ± 13.50 ms) for both tasks for two groups, *p* = 0.046, *p* = 0.001, *p* < 0.001, *p* = 0.004, η^2^ = 0.06, 0.16, 0.18, 0.12. There are also revealed that the response time of incongruent trials was longer than congruent trials for two groups for both pre-test and post-test, *p*s < 0.001, η^2^ = 0.80, 0.77, 0.73, 0.77. But the response time of experiment and control groups didn’t have a significant difference for pre-test and post-test for both tasks, *p* = 0.54, 0.76, 0.63, 0.78. No other significant main effect or two-way interactions were observed.

### ERP Data

**FIGURE 3:**
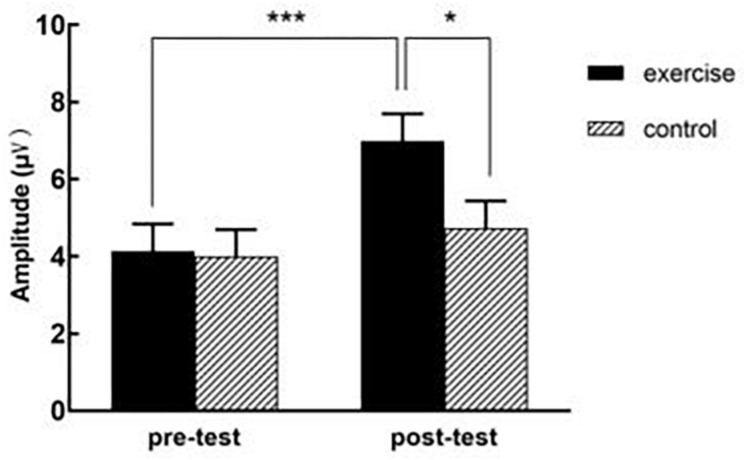
The interaction effect of the P2 amplitude between time × treatment. ^∗∗∗^represent *p* value < 0.001, ^∗^represent 0.01 < *P* < 0.05.

**FIGURE 4:**
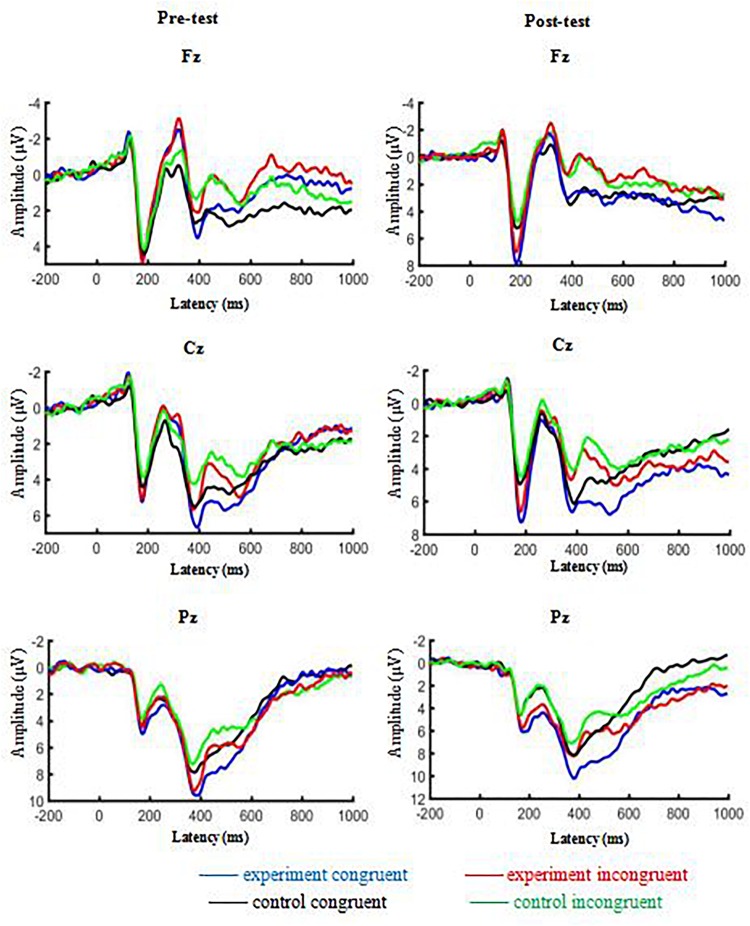
Grand-averaged ERP waveforms as a function of treatments and Stroop tasks.

**FIGURE 5:**
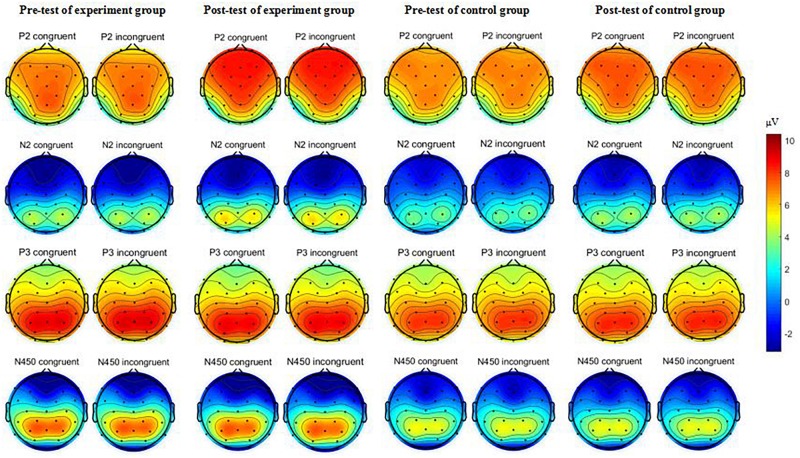
The topographic scalp distributions of the P2, N2, P3b, and N450 components.

A datum of the experiment group dropped out because of recording failed. Table 3 presents ERP values for each condition, and Table 4 provides a detailed statistical summary table for significant effects. Figure 3 illustrates the interation effect of the P2 amplitude between time × treatment. Figure 4 illustrates the grand-averaged ERP waveform for each treatment and task, and Figure 5 illustrates the topographic scalp distribution of the P2, N2, P3b, and N450 components.

**TABLE 3:** ERP amplitude and latency data (mean ± SE) across treatments and Stroop tasks.

**Component**	**Experiment group**				**Control group**			
	**Pre-test**		**Post-test**		**Pre-test**		**Post-test**	
	**Congruent**	**Incongruent**	**Congruent**	**Incongruent**	**Congruent**	**Incongruent**	**Congruent**	**Incongruent**
**Amplitude (μV)**								
P2	4.14 ± 0.69	4.14 ± 0.74	7.45 ± 0.68	6.53 ± 0.78	4.15 ± 0.68	3.86 ± 0.73	5.02 ± 0.67	4.46 ± 0.77
N2	−2.13 ± 0.60	−2.56 ± 0.63	−1.41 ± 0.76	−2.06 ± 0.72	0.05 ± 0.59	−0.89 ± 0.62	−0.47 ± 0.75	−1.49 ± 0.71
P3b	9.25 ± 0.85	8.82 ± 0.84	9.90 ± 0.94	7.99 ± 0.78	7.69 ± 0.84	7.03 ± 0.83	7.92 ± 0.93	6.93 ± 0.77
N450	1.87 ± 0.72	0.27 ± 0.74	2.57 ± 0.89	0.04 ± 0.80	2.15 ± 0.71	0.34 ± 0.73	2.45 ± 0.88	0.22 ± 0.78
**Latency (ms)**								
P2	186.40 ± 3.60	186.97 ± 3.84	184.57 ± 3.29	185.37 ± 3.68	187.89 ± 3.55	187.44 ± 3.79	189.44 ± 3.24	190.44 ± 3.63
N2	302.06 ± 5.23	300.11 ± 5.63	303.31 ± 5.19	306.40 ± 5.00	298.56 ± 5.16	302.11 ± 5.55	297.78 ± 5.12	300.67 ± 4.93
P3b	382.40 ± 4.04	380.11 ± 3.70	379.20 ± 4.41	376.23 ± 3.84	379.89 ± 3.98	381.22 ± 3.64	385.11 ± 4.35	375.78 ± 3.79
N450	472.57 ± 6.08	467.77 ± 5.24	459.54 ± 6.26	464.11 ± 6.01	464.89 ± 5.99	469.11 ± 5.16	464.22 ± 6.17	463.33 ± 5.92

**TABLE 4:** Statistical summary table for behavioral and ERP data.

**Measure**	**Effect**	**df**	***F***	***p***	**Partial η^2^**
Accuracy	Time (post-test > pre-test)	1,70	43.4	< 0.001	0.38
	**Task (congruent > incongruent)**	**1,70**	**76.3**	**< 0.001**	**0.52**
	Task × time	1,70	34.22	< 0.001	0.33
RT	Time (pre-test > post-test)	1,70	23.63	< 0.001	0.25
	**Task (incongruent > congruent)**	**1,70**	**642.66**	**< 0.001**	**0.90**
	Treatment × task × time	1,70	4.4	0.04	0.06
P2 amplitude	**Task (congruent > incongruent)**	**1,69**	**5.63**	**0.02**	**0.08**
	Time (post-test > pre-test)	1,69	27.74	< 0.001	0.29
	**Time × treatment**	**1,69**	**9.67**	**0.003**	**0.12**
N2 amplitude	**Task (incongruent > congruent)**	**1,69**	**10.89**	**0.002**	**0.14**
P3b amplitude	**Task (congruent > incongruent)**	**1,69**	**17.56**	**< 0.001**	**0.20**
	Task × time	1,69	4.43	0.04	0.06
N450 amplitude	**Task (incongruent > congruent)**	**1,69**	**46.9**	**< 0.001**	**0.41**

*Only significant effects at *p* < 0.05 are presented; Only bold the main findings.*

#### Analysis

##### P2 component

For amplitude, the three-way ANOVA revealed significant main effects for task, *F*(1, 69) = 5.63, *p* = 0.02, partial η^2^ = 0.08, with congruent task (5.18 ± 0.45 μV) yielding larger amplitude than incongruent task (4.74 ± 0.49 μV), and for time, *F*(1, 69) = 27.74, *p* < 0.001, partial η^2^ = 0.29, with post-test (5.87 ± 0.50 μV) yielding larger amplitude than pre-test (4.07 ± 0.49 μV). And there was a significant two-way interaction between time × treatment, *F*(1, 69) = 9.67, *p* = 0.003, partial η^2^ = 0.12. Follow-up analyses revealed that there was no significant difference between experiment group (4.14 ± 0.70 μV) and control group (4.01 ± 0.69 μV) for pre-test, *p* = 0.89, but experiment group (6.99 ± 0.71 μV) yielded a larger amplitude than control group (4.74 ± 0.70 μV) for post-test, *p* = 0.026, partial η^2^ = 0.07. The analyses also revealed that post-test yielded larger amplitude than pre-test in the experiment group, *p* < 0.001, partial η^2^ = 0.33, but the amplitude of pre-test and post-test didn’t show a significant difference in the control group, *p* = 0.13. No significant main effect for treatment, two-way or three-way interactions were observed.

In terms of P2 latency, no significant main effects, two-way or three-way interactions were observed.

##### N2 component

For amplitude, the three-way ANOVA revealed significant main effects for task, *F*(1, 69) = 10.89, *p* = 0.002, partial η^2^ = 0.14, with incongruent task (−1.75 ± 0.43 μV) yielding more negative amplitude than congruent task (−0.99 ± 0.44 μV). No other significant main effects, two-way or three-way interactions were observed.

In terms of N2 latency, no significant main effects, two-way or three-way interactions were observed.

##### P3b component

For amplitude, the three-way ANOVA revealed significant main effects for task, *F*(1, 69) = 17.56, *p* < 0.001, partial η^2^ = 0.20, with congruent task (8.69 ± 0.57 μV) yielding larger amplitude than incongruent task (7.69 ± 0.52 μV). And there was a significant two-way interaction between task × time, *F*(1, 69) = 4.43, *p* = 0.039, partial η^2^ = 0.06. Follow-up analyses revealed that congruent task (8.47 ± 0.60 μV, 8.91 ± 0.66 μV) yielded larger amplitude than incongruent task (7.92 ± 0.60 μV, 7.46 ± 0.55 μV) for both pre-test and post-test, *p* = 0.041, *p* < 0.001, partial η^2^ = 0.06, 0.18, and the analyses also revealed that the amplitude between pre-test and post-test didn’t have significant difference for congruent task as well as incongruent task, *p* = 0.43, *p* = 0.30. No other significant main effects, two-way or three-way interactions were observed.

In terms of P3b latency, no significant main effects, two-way or three-way interactions were observed.

##### N450 component

For amplitude, the three-way ANOVA revealed significant main effects for task, *F*(1, 69) = 46.90, *p* < 0.001, partial η^2^ = 0.41, with incongruent task (0.22 ± 0.49 μV) yielding more negative amplitude than congruent task (2.26 ± 0.51 μV). No other significant main effects, two-way or three-way interactions were observed.

In terms of N450 latency, no significant main effects, two-way or three-way interactions were observed.

## Discussion

The purpose of this study was to investigate whether acute moderate-intensity aerobic exercise for approximate 25 min led to a selective effect on executive function tasks or general effect on cognitive tasks that involve executive function and basic information processing in young adults. Besides, we also aimed to expand previous research by examining multiple ERP components (e.g., P2, N2, P3b, and N450). In our study, we found acute exercise led to larger P2 amplitudes for both congruent and incongruent Stroop tasks. The current findings indicate that acute moderate-intensity aerobic exercise may have a beneficial effect on mobilization of attentional resources related to perceptual processing and exercise-related physiological arousal.

### Task Performance

This study found that compared with congruent task, the longer response time and lower accuracy of incongruent task reflected the typical “Stroop effect” (Ridley Stroop, 1992) which supported the Stroop task design we used in this study and indicated that incongruent task needed a greater amount of inhibitory cognitive control (Chang et al., 2014c, 2017). Regarding the effect of acute exercise on the accuracy, although the accuracy of post-test was higher than pre-test, the promotion didn’t show significant difference between experimental and control groups which suggested that the promotion just resulted from practice effect rather than acute exercise. As to the response time, experiment group and control group didn’t show significant difference for both pre-test and post-test, so we didn’t find the effect of acute exercise on response time, either.

### P2 Component

In this study, compared with the congruent task, the smaller P2 amplitude of incongruent task in the frontal-central region replicated findings from previous studies (Yuan et al., 2011; Zinchenko et al., 2017).

P2 component has been related to bottom-up or low-level processing of information, such as stimulus classification and categorization (Crowley and Colrain, 2004). A smaller P2 amplitude for the incongruent task might indicate reduced information gained from stimuli (Zinchenko et al., 2014). Although acute exercise did not influence P2 latency, an enhanced P2 amplitude was observed for the incongruent task following acute exercise versus control. The magnitude of frontal P2 activity has been associated with an index of the intensity of perceptual processing which requires attention allocation to function (Chen et al., 2007; Yuan et al., 2007, 2012). Although the primary emphasis in the inhibition literature has been the N2/P3b complex, earlier components such as the P2 component may also play an important role in inhibition success (Thomas et al., 2009; Benikos et al., 2013). These results might imply that acute exercise allocated more attentional resources to protect against interference from irrelevant stimuli (Garcia-Larrea et al., 1992), giving the imperative stimulus a clear path for further processing (Oades, 1998). However, a similar enhancement in P2 amplitude was observed for the congruent task, suggesting that acute exercise might also mobilize more attentional resources related to basic information processing. Notably, the P2 component is sensitive to the arousal level (Schaefer et al., 2011; Feng et al., 2014; Imbir et al., 2017). And an inverted-U profile (McMorris and Hale, 2012) suggests the facilitative effect of acute exercise on cognitive function is obtained when arousal is induced by moderate-intensity exercise, the current findings further supported a physiological arousal hypothesis of acute exercise’s effects on generalized cognitive performance. Collectively, the evidence suggested that acute moderate-intensity aerobic exercise might enhance attentional resources related to perceptual processing, and increased exercise-related physiological arousal was associated with P2 amplitude, regardless of Stroop task types. Interestingly, modulation of the P2 amplitude has not been connected with acute moderate-intensity exercise, so the hypothesis was not formulated *a priori*. That is to say, this is the first study to observe a significant effect of acute moderate-intensity exercise on the modulation of the P2 amplitude.

### Other ERP Components

Several other stimulus-locked ERP components elicited by the Stroop task were also examined. For example, a larger N2 amplitude was observed for Stroop incongruent trials than congruent trials in the frontal-central region which might indicate incongruent task required a greater recruitment of cognitive resources for conflict monitoring (Botvinick et al., 2004; Veen et al., 2007; Gu et al., 2019) which replicated findings from previous studies (Larson et al., 2014; Ligeza et al., 2018; Gu et al., 2019). Compared with the congruent task, a larger N450 amplitude for incongruent task might reflect monitoring processes involved in conflict detection (Badzakova-Trajkov et al., 2009; Larson et al., 2014) which replicated previous findings (Chang et al., 2017). A smaller P3b amplitude was observed for incongruent task compared with congruent task in the central-parietal region which replicated findings from previous studies (Ila and Polich, 1999; Chang et al., 2017). The P3b component elicited by the Stroop task has been associated with cognitive processes related to semantic conflict (Chang et al., 2017). This finding might imply the experience of greater semantic conflict and task difficulty during the performance of incongruent task. Our findings showed no effect of acute moderate-intensity exercise on the P3b component. Previous studies usually reported modulation of P3b by acute exercise (Kamijo et al., 2009; O’Leary et al., 2011; Chu et al., 2017), but some studies also showed no effect of acute exercise on the P3b component (Ligeza et al., 2018). In our study, the possible explanation might be the population we chose, a previous meta-analysis indicated acute exercise showed larger positive effects on cognitive performance in adolescents and older adults than young adults (Chang et al., 2012). Unlike adolescents’ cognitive function is at the stage of development or cognitive function of elderly people is declining, cognitive function of university students is relatively stable and mature, so acute exercise showed smaller effects on cognitive performance in young adults.

### Limitations

Previous studies usually reported modulation of the P3b component by acute exercise, but this study failed to find the effect of acute exercise on the P3b component. It may result from the population we chose, acute exercise shows smaller effects on cognitive performance in young adults (Chang et al., 2012).

## Conclusion

It is the first study to find acute moderate-intensity aerobic exercise generally affects early information processes through greater mobilization of attentional resources related to perceptual processing and an increase in exercise-related physiological arousal reflected by the P2 amplitude.

## Data Availability Statement

The datasets generated for this study are available on request to the corresponding author.

## Ethics Statement

The studies involving human participants were reviewed and approved by the ethics committee of the Southwest University. The patients/participants provided their written informed consent to participate in this study.

## Author Contributions

Both authors listed have made a substantial, direct and intellectual contribution to the work, and approved it for publication.

## Conflict of Interest

The authors declare that the research was conducted in the absence of any commercial or financial relationships that could be construed as a potential conflict of interest.
